# Supplementary material for: Hybrid sample size calculations for cluster randomised trials using assurance
Source: Clin Trials. 2025 Feb 11;22(5):517–26. doi: 10.1177/17407745241312635 (PMC12476461; doi:10.1177/17407745241312635)
Supplement: sj-pdf-1-ctj-10.1177_17407745241312635 – Supplemental material for Hybrid sample size calculations for cluster randomised trials using assurance [file sj-pdf-1-ctj-10.1177_17407745241312635.pdf]

Hybrid sample size calculations for cluster randomised trials using assurance: Supplemental Material

S. Faye Williamson[1]*, Svetlana V. Tishkovskaya[2], Kevin J. Wilson[3]

[1] Biostatistics Research Group, Population Health Sciences Institute, Newcastle University, UK

[2] Faculty of Health and Care, Lancashire Clinical Trials Unit, University of Central Lancashire, UK

[3] School of Mathematics, Statistics & Physics, Newcastle University, UK

November 25, 2024

1 Appendix

1.1 Simulation study

In this section, we conduct a more general investigation of the properties of the hybrid approach compared to the standard power calculation. We use a significance level of $\alpha = 0.05$ and a power of 0.9 for a one-sided Wald test for the treatment effect. The true values of the model parameters are as follows: the overall standard deviation $\sigma = 1$, the treatment effect $\delta = 0.5$, the coefficient of variation in the sample size $\nu = 0.5$ and the ICC $\rho = 0.05$. Suppose we have $J = 20$ clusters. For the assurance calculations, we choose a prior variance for ρ of 0.02, for ν of 0.01 and vary the prior variance of σ between 0.1 and 0.5. This represents a situation where we have some prior knowledge about the nuisance parameters, but we still have uncertainty as to their true values. The values of σ_b^2 and σ_w^2 are calculated from ρ and σ as $\sigma_b^2 = \sigma^2/[1+\rho/(1-\rho)] = 0.8$ and $\sigma_w^2 = \sigma^2/[1+(1-\rho)/\rho] = 0.2$, respectively.

The simulation structure is as follows. First, we calculate the total sample size N, using the desired

*Corresponding author. Email: faye.williamson@newcastle.ac.uk

power or assurance. This gives the average sample size per cluster, $\bar{n}$. Then, for each of 10,000 samples, we:

1. Simulate sample sizes $n_1, \ldots, n_{J-1}$ from $n_j \sim \mathrm{N}(\bar{n}, \bar{n}\nu)$ and set $n_J = N - \sum_{j=1}^{J-1} n_j$. The first $J/2$ clusters are assumed to be treatment and the rest control.

2. For individual i in cluster j, simulate $e_{ij} \sim \mathrm{N}(0, \sigma_w^2)$ and for each cluster j, simulate a cluster effect $c_j \sim \mathrm{N}(0, \sigma_b^2)$.

3. Using these simulated values, calculate the responses y_{ij} for individual i in cluster j using (1) in the main paper.

4. Fit the linear mixed-effects model in (1) from the main paper to the responses using the `lmer` function in `R` [Bates et al., 2015].

5. Extract the treatment effect estimate $\hat{\delta}$ and its corresponding test statistic T.

Comparing the 10,000 test statistics for δ to their corresponding critical value provides an estimate of the type II error rate. Repeating steps 3 and 4 with $\delta = 0$ gives an estimate of the type I error rate. That is, the Wald test is conservative in this case.

We use the true parameter values for the power calculation and centre the prior distributions at the true parameter values for the assurance calculations. The resulting average cluster sample sizes $\bar{n}$, estimates of δ, T, type I and type II error rates, based on power and assurance calculations with a prior standard deviation on σ of $s_\sigma = (0.1, 0.2, 0.3, 0.4, 0.5)$, are provided in Table 1. Boxplots of $\hat{\delta}$ and the values of T over the 10,000 simulations are shown in Figure 1.

Approach	$\bar{n}$	E[$\hat{\delta}$]	SD($\hat{\delta}$)	E[T]	SD(T)	Type II	Type I
Power	12	0.50	0.16	3.17	1.17	0.14	0.03
Assurance ($s_\sigma = 0.1$)	12	0.50	0.16	3.16	1.16	0.14	0.03
Assurance ($s_\sigma = 0.2$)	14	0.50	0.16	3.34	1.19	0.11	0.03
Assurance ($s_\sigma = 0.3$)	16	0.50	0.15	3.49	1.21	0.09	0.03
Assurance ($s_\sigma = 0.4$)	19	0.50	0.14	3.64	1.23	0.07	0.04
Assurance ($s_\sigma = 0.5$)	24	0.50	0.14	3.85	1.26	0.05	0.03

Table 1: The average sample size per cluster $\bar{n}$, the mean and standard deviation of $\hat{\delta}$, the mean and standard deviation of the test statistic T, and the type I and II error rates over 10,000 simulations, using power and assurance with different standard deviations s_σ on the prior for σ.

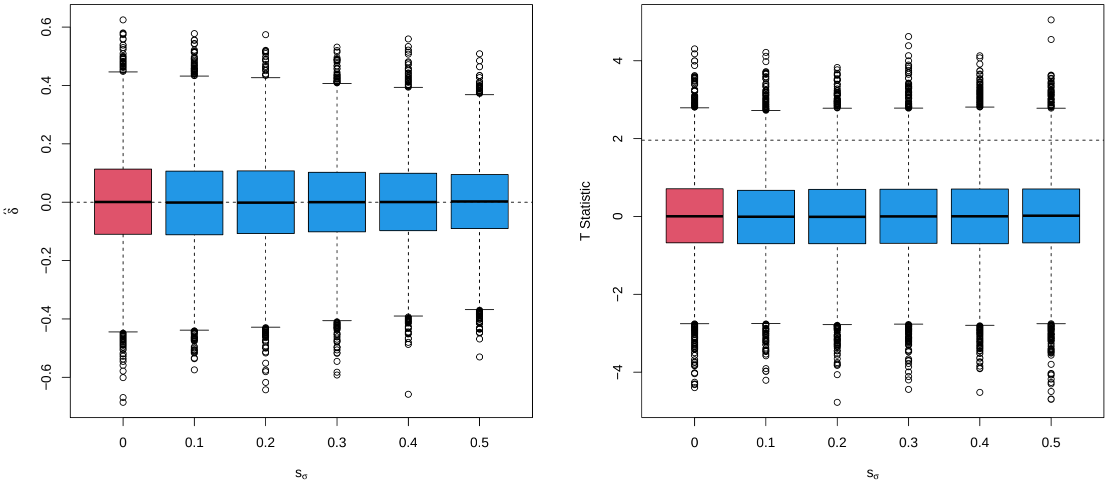

Figure 1: Boxplots of the estimates of δ (left) and T (right) for the 10,000 simulations, based on the sample sizes chosen using power (red) and assurance (blue) with standard deviations on σ of (left to right in each plot) 0.1, 0.2, 0.3, 0.4 and 0.5. The power calculation was conducted using the true parameter values, and the assurance priors were centred at the true values.

Table 1 shows that the average sample size per cluster, based on the assurance, increases as the standard deviation on the prior for σ, s_σ, increases. When $s_\sigma = 0.1$, representing a very informative prior, the sample size is the same as that for power. All of the sample sizes result in unbiased estimators for δ, with larger sample sizes reducing the variability in the estimates. The value of the test statistic increases on average with larger sample sizes, with a small increase in variability. Based on the power calculation, the type II error rate is a little higher than would provide nominal 90% power, and the type I error rate is a little lower than the desired significance level of 5%.

1.1.1 Misspecification of σ

In Section 3.3 of the main paper we considered the effect of misspecifying ρ. In this section, we investigate the effect of misspecifying σ on the resulting power/assurance of the hypothesis test for δ. We vary $\hat{\sigma}$, which is used for the power calculation and as the prior mean for the assurance calculation, in the interval $[0.8, 1.5]$ and illustrate the resulting power based on 10,000 simulations in the left hand side of Figure 2.

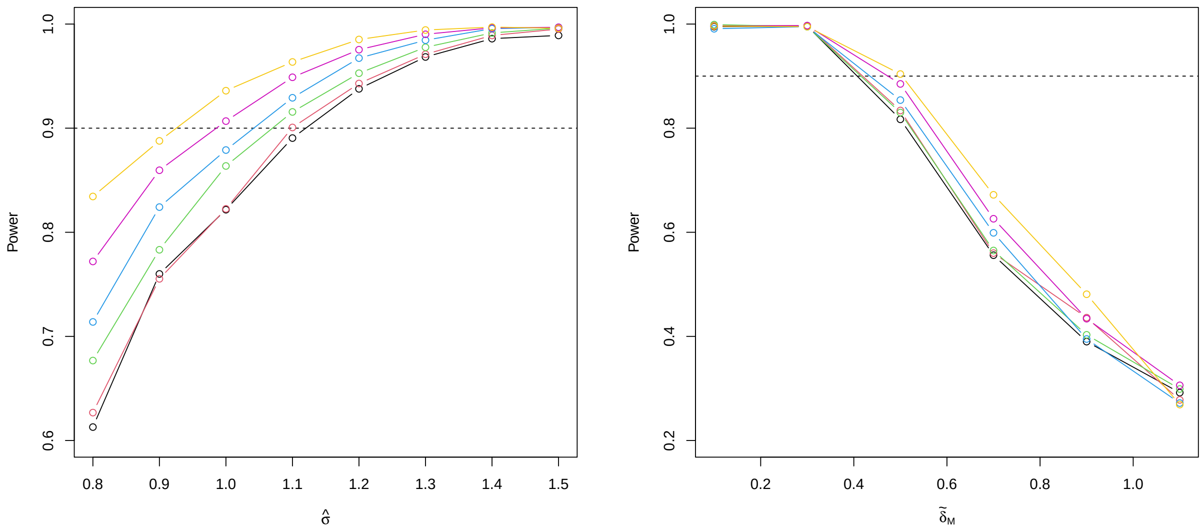

Figure 2: The effect of misspecifying σ (left) and different values for the MCID δ_M (right) on the resulting power, estimated over 10,000 simulations. The black line corresponds to the power obtained using a sample size chosen using a power calculation. The coloured lines represent the power obtained using a sample size chosen using an assurance calculation with $s_\sigma = 0.1$ (red), $s_\sigma = 0.2$ (green), $s_\sigma = 0.3$ (blue), $s_\sigma = 0.4$ (pink) and $s_\sigma = 0.5$ (yellow).

The power calculation (black line) is the most sensitive to changes in the estimated value of σ, with a misspecification of just 0.2 (e.g. choosing $\hat{\sigma} = 0.8$) resulting in a power of only 65%. As the standard deviation on the prior for σ increases, that is, the more uncertainty we have in the true value of σ, the more robust the chosen sample size is to misspecification. With $s_\sigma = 0.5$ (yellow line), we retain over 80% power with the prior centred on the incorrect value of $\hat{\sigma} = 0.8$.

Figure 2 shows that if there is no good quality information available on σ, it may be preferable to use assurance to obtain a more robust sample size because this gives an expected power closer to the nominal value of 90%.

1.1.2 Effect of varying the MCID δ_M

The value chosen for the MCID δ_M may be context specific. For example, we may choose a low MCID for a treatment that is cheaper, or has fewer side effects, than the standard of care, and a higher MCID for a treatment with similar costs to the standard of care. Consequently, the MCID may not be fixed

for different treatments, or the same treatment at different stages of a trial (as we learn more about it). Therefore, we investigate the effect of varying the MCID on the properties of the sample size chosen using power and assurance.

We retain the true MCID as $\delta_M = 0.5$ and vary the value used for the power or assurance calculation $\tilde{\delta}_M$ in the interval $[0.1, 1.1]$. We show the power of the resulting sample size, based on 10,000 simulations, in the right hand side of Figure 2. The values of $\tilde{\delta}_M$ are plotted against the simulated power, based on sample sizes chosen using power (black) and assurance with s_σ of 0.1 (red), 0.2 (green), 0.3 (blue), 0.4 (pink) and 0.5 (yellow).

We observe similar effects to when we varied $\hat{\sigma}$. When the value used for the MCID is much smaller than 0.5, the sample size chosen will be larger than necessary and we obtain higher power than required. As the MCID becomes greater than 0.5, the sample size chosen is too small to yield adequate power. This loss of power is strongest for the power calculation and assurance with a small standard deviation on σ, and decreases as the standard deviation on σ increases.

References

Douglas Bates, Martin Mächler, Ben Bolker, and Steve Walker. Fitting linear mixed-effects models using lme4. *Journal of Statistical Software*, 67(1):1–48, 2015. doi: 10.18637/jss.v067.i01.
